# Species of the fungivorous genus *Psalidothrips* Priesner from China, with five new species (Thysanoptera, Phlaeothripidae)

**DOI:** 10.3897/zookeys.746.22882

**Published:** 2018-03-26

**Authors:** Chao Zhao, Hongrui Zhang, Xiaoli Tong

**Affiliations:** 1 Department of Entomology, College of Agriculture, South China Agricultural University, Guangzhou 510642, China; 2 Plant Protection College, Yunnan Agricultural University, Kunming 650201, China

**Keywords:** fungus-feeding, leaf-litter thrips, new species, Phlaeothripinae

## Abstract

An identification key and review is provided of fifteen species of the fungivorous genus *Psalidothrips* Priesner from China, with five new species, *P. angustus* **sp. n.**, *P. comosus* **sp. n.**, *P. fabarius* **sp. n**., and *P. latizonus* **sp. n**., and *P. nigroterminatus* **sp. n.** In addition, *Psalidothrips consimilis* Okajima, previously known only from Ryukyu Islands, Japan, is newly recorded in China.

## Introduction

*Psalidothrips* Priesner is one of the most common phlaeothripine genera in tropical and subtropical regions. The members of the genus are fungus-feeders, and are particularly associated with leaf litter, their body and wing form probably being an adaptation to such habitats (Mound 1970; Okajima 1983). The genus appears to be derived from *Hoplothrips* and belongs to the *Phlaeothrips*-lineage (Mound and Marullo 1996; Okajima 2006; Dang et al. 2014). Okajima (1983) reviewed the genus worldwide and provided an identification key to 17 known species. Subsequently, eleven further species were added to the genus: from New Zealand (three species), Japan (four species), and China (four species) (respectively Mound and Walker 1986; Okajima 1992; Zhang and Tong 1997; Wang et al. 2007). Okajima and Urushihara (1992) transferred *Trichothrips lewisi* Bagnall, to *Psalidothrips* and treated *P. alaris* Haga as a synonym of *P. lewisi* (Bagnall). Later, *P. lepidus* zur Strassen was considered a synonym of *P. conciliatus* Hood, and *Hennigithrips ananthakrishnani* Johansen was transferred to *Psalidothrips* by Mound and Marullo (1996). Up to the present, 28 *Psalidothrips* species are known worldwide, 15 of these being from Asia (ThripsWiki 2017). The purpose of this paper is to review the *Psalidothrips* species now recognized from China, to provide an updated identification key to these 15 species, including five new species and one newly recorded species from China, together with male pore plate illustrations of thirteen species.

## Materials and methods

All thrips specimens in this study were extracted by using Tullgren funnels from leaf litter unless otherwise noted, and the specimens then were sorted and preserved in 90% alcohol. Examined specimens were mounted in Canada balsam using the method outlined by Zhang et al. (2006). Slide-mounted specimens were examined and photographed under the microscope with a digital camera attached. The following abbreviations are used for the pronotal setae:

**am** anteromarginal

**aa** anteroangular

**ml** midlateral

**epim** epimeral

**pa** posteroangular

Slide-mounted specimens of *P. lewisi* (male and female), *P. longiceps*, and *P. simplus* (male and female) have also been examined; these were provided by Professor Okajima of Tokyo University of Agriculture (**TUA**, Japan). All type specimens are preserved in the Insect Collection, South China Agricultural University (**SCAU**) unless otherwise noted.

## Taxonomy

### Key to *Psalidothrips* species from China

**Table:** 

1	Antennal segment III with 2 sense cones	**2**
–	Antennal segment III with 3 sense cones	**6**
2	Abdominal tergites III to VII each with one pair of simple wing-retaining setae; antennal segment IV with 2–4 sense cones; abdominal tergite II concolourous with the other tergites; pelta trapezoidal or broadly hat-shaped; postocellar setae much shorter than hind ocellus; male pore plate transversely long oval (Fig. 78)	***simplus***
–	Abdominal tergites III to VII each with two pairs of wing-retaining setae; antennal segment IV with 2 sense cones only	**3**
3	Head largely yellow	**4**
–	Head uniformly brown	**5**
4	Antennal segments I–III yellow, IV–VIII dark brown; abdominal tergite II concolourous with the other tergites; male pore plate (Fig. 59) arched with slightly straight anterior margin	***nigroterminatus* sp. n.**
–	Antennal segments I–II pale brown, III–VIII gradually from yellow to pale brown towards apex; abdominal tergite II darker than other tergites; male pore plate broad and arched, reaching to lateral margins (Fig. 70)	***latizonus* sp. n.**
5	Antennal segments I–II and VI–VIII brown, other segments yellow; postocellar setae slightly longer than hind ocellus; postocular setae slightly longer than eyes and pointed at apex; antennal segments IV–VI not globular; male pore plate narrow and slightly arched (Fig. 73)	***chebalingicus***
–	Antennal segments I and basal half of II light brown, segment III yellow, IV–VIII yellowish brown, gradually darkened distally; postocellar setae twice as long as hind ocellus; postocular setae approximately 1.5 times longer than eyes, blunt or weakly expanded at apex; antennal segments IV–VI globular; male pore plate arched with a projection medially (Fig. 69)	***fabarius* sp. n.**
6	Antennal segment IV with 3 sense cones	**7**
–	Antennal segment IV with 4 sense cones	**11**
7	Fore tarsal tooth present in both sexes	**8**
–	Fore tarsal tooth absent in female	**9**
8	Postocular setae blunt apically; antennal segment VIII longer than segment VII; pelta irregularly trapezoidal without campaniform sensilla; male pore plate slightly arched (Fig. 77)	***longidens***
–	Postocular setae expanded apically; antennal segment VIII as long as segment VII; pelta hat-shaped with a pair of campaniform sensilla; male unknown	***armatus***
9	Postocular setae longer than eyes	**10**
–	Postocular setae shorter than eyes with apices acute; antennal segments brown except III–IV yellow, segment VIII longer than VII; male pore plate arched and reaching to near the margins (Fig. 72)	***bicoloratus***
10	Postocular setae and pronotal epim with apices expanded; sense cones on antennal segment IV about two thirds as long as the segment; abdominal tergite II concolourous with other tergites; male unknown	***amens***
–	Postocular setae and pronotal epim with apices pointed; sense cones on antennal segment IV about half as long as the segment; abdominal tergite II darker than other tergites; male pore plate shuttle-shaped (Fig. 75)	***elagatus***
11	Fore tarsal tooth present in female	**12**
–	Fore tarsal tooth absent in female	**13**
12	Antennae brown except basal third of III yellowish brown, surface without sculpture; postocular setae expanded at apex; abdominal tergites II to VII each with two pairs of wing-retaining setae; male pore plate slightly arched and incomplete, not reaching lateral margins (Fig. 67)	***angustus* sp. n.**
–	Antennae almost uniformly yellow, segments III–VII with lines of sculpture; postocular setae pointed at apex; abdominal tergites II to VII each with one pair of wing-retaining setae; male pore plate on abdominal sternite VIII narrow and arched, reaching to near lateral margins (Fig. 68)	***comosus* sp. n.**
13	Postocellar setae much longer than diameter of hind ocellus; antennal segment VIII as long as segment VII; male pore plate banded and complete, reaching lateral margins (Fig. 76)	***lewisi***
–	Postocellar setae usually as long as or shorter than diameter of hind ocellus	**14**
14	Antennal segment VIII slightly longer than segment VII, segments III and IV somewhat globular; male pore plate arched and incomplete (Fig. 74)	***consimilis***
–	Antennal segment VIII as long as or shorter than segment VII, segments III and IV slender, not globular; male pore plate arched and complete (Fig. 71)	***ascitus***

#### Psalidothrips amens

Taxon classificationAnimaliaThysanopteraPhlaeothripidae

Priesner

Psalidothrips amens Priesner, 1932: 62.

##### Comments.

This is the type species of the genus, and was based on a single female from Java, Indonesia. It belongs to the group that have antennal segments III–IV each with three sense cones. Two females listed here are identified as *P. amens* based on the original description and the key in Mound (1970). Wang et al. (2007) mentioned that a single male of *P. amens* was recorded from Hainan, China. We examined that slide-mounted specimen, labelled as the male of *P. amens* by Wang et al. (2007), and consider that it represents the male of *P. latizonus* sp. n., described below. In China, *P. amens* is found, so far, only from Hainan.

##### Distribution.

China (Hainan); Indonesia (Java).

#### Psalidothrips angustus sp. n.

Taxon classificationAnimaliaThysanopteraPhlaeothripidae

http://zoobank.org/14F2A513-EDFD-4FD2-ADE8-75A00A92B4EC

Figs 1–2 , 29–36 , 67

##### Material examined.

**Holotype** female: **CHINA**, **Guangdong**: Guangzhou, Arboretum of South China Agricultural University (23°09'22"N, 113°21'15"E), 10.x.2014 (Chao Zhao).

##### Paratypes.

Four females and 1 male, collected with holotype; 42 females and 14 males, same locality as holotype, 29.xii.2013 (Jingna Li), 5 females and 1 male, 14.vii.2014 (Chao Zhao). **Guangdong**: Guangzhou City, Longdong (23°14'N, 113°24'E), 1 female, 1.xii.2006 (Jun Wang); Panyu, Dafushan Forest Park (22°57'33"N, 113°18'0"E), 2 females, 10.x.2014 (Chao Zhao). **Hainan**: Ledong County, Jianfengling National Nature Reserve (18°44'N, 108°51'E), 1 female, 31.x.1986 (Xiaoli Tong); Qiongzhong County, Limushan National Forest Park (19°12'40"N, 113°12'39"E, alt. 1200 m), 3 females and 1 male, 24.x.2017 (Chao Zhao).

##### Description.

**Figures 1–4.**
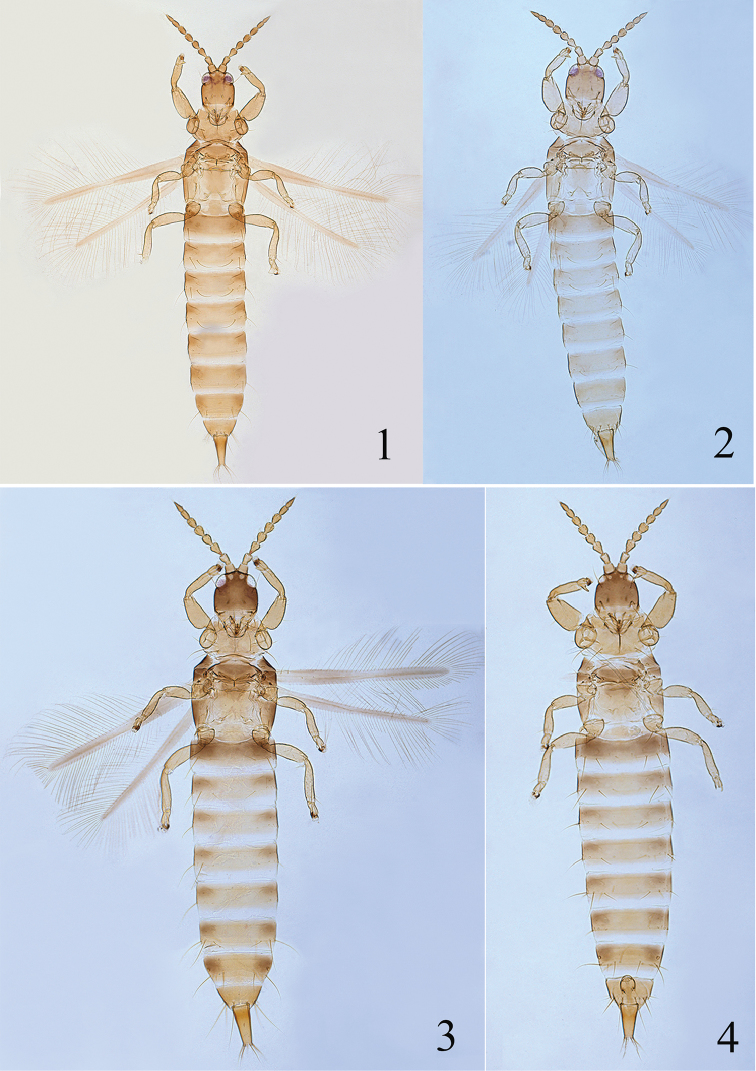
New *Psalidothrips* species. *Psalidothrips angustus* sp. n.: **1** female **2** male; *Psalidothrips comosus* sp. n. **3** female **4** male.

**Female macropterous** (Fig. 1). Head and antennae brown (but basal third of segment III paler), pronotum pale brown; mesonotum, abdominal segment II and sides of tergites III–VIII brown; the rest of body yellow or yellowish brown; fore wings greyish brown but paler medially.

*Head* almost as long as broad; dorsal surface smooth, faintly sculptured posteriorly; cheeks almost straight and constricted behind eyes. Eyes approximately one-third of head length; postocular setae much longer than eyes, expanded at apex (Fig. 29); postocellar setae fine and acute, longer than diameter of hind ocellus. Antennae 8-segmented (Fig. 36), somewhat moniliform, surface without sculpture; segment III vasiform and IV–V globular, segment VIII longer than segment VII; segments III–IV with three and four sense cones respectively, sense cones usually long and thick, those on segment IV usually longer than half of the segment. Maxillary stylets reaching approximately half way to postocular setae and placed far apart, often V-shaped.

*Pronotum* broad (Fig. 30), dorsal surface smooth with a weak median longitudinal line; pronotal am and aa setae minute; ml, epim, and pa setae well developed, ml with expanded apex, epim and pa bluntly acute. Mesonotum sculptured on anterior third, lateral setae minute. Metanotum largely smooth with faint sculpture laterally. Mesopresternum boat-shaped, often eroded medially (Fig. 31). Fore tarsal tooth present (Fig. 32). Fore wing wide at base and constricted medially, sub-basal setae S1 minute, S2 slightly longer than S3.

*Pelta* hat-shaped with flat anterior margin, faintly reticulate medially, a pair of campaniform sensilla present (Fig. 34). Abdominal tergites II–VII with two pairs of weakly sigmoid wing-retaining setae; tergite IX setae S1 subequal to tube in length and shorter than S2; S2 slightly longer than tube, both pointed at apex (Fig. 35).

*Measurements* (holotype female in microns). Distended body length 1890. Head length 195, width 175; eye length 65; postocular setae length 80; diameter of posterior ocellus 22; postocellar setae length 32. Antennal length 360, segments I–VIII length (width) as follows: 36 (42); 40 (30); 55 (35); 48 (37); 43 (33); 42 (30); 38 (22); 46 (35). Pronotum median length 145, median width 270; length of major setae: ml 70, pa 85, epim 75. Fore wing length 800, subbasal setae S2–S3 length: 22, 15. Abdominal tergite IX S1 setae length 145, S2 setae length 165. Tube length 150, basal width 75, apical width 32; anals 140.

**Male macropterous** (Fig. 2). Similar in colour and structure to female, but body smaller; fore tarsal tooth present (Fig. 33); pore plate on abdominal sternite VIII disconnected and slightly arched (Fig. 67); abdominal tergite IX setae S1 as long as tube and longer than S2.

*Measurements* (paratype male in microns). Distended body length 1450. Head length 170, width 160; eye length 55; postocular setae length 65; diameter of posterior ocellus 15; postocellar setae length 25. Antennal length 310, segments I–VIII length (width) as follows: 31 (40); 32 (27); 46 (28); 38 (29); 38 (28); 37 (25); 32 (22); 40 (18). Pronotum median length 135, median width 260; length of major setae: ml 60, pa 75, epim 57. Fore wing length 520, subbasal setae S2–S3 length: 13, 21. Abdominal tergite IX S1 setae length 100, S2 setae length 75. Tube length 120, basal width 80, apical width 20, anals 105.

##### Etymology.

The specific epithet, *angustus*, is from the Latin adjective, meaning narrow and refers to the narrow pore plate.

##### Distribution.

China (Guangdong, Hainan).

##### Comments.

This new species appears to be closely related to *P. comosus* sp. n., by sharing moniliform antennae and antennal segments III–IV with three and four sense cones, and the fore tarsal tooth present in female. However, it differs from the latter by the following characteristics: (1) the surface of antennae is without sculpture (apical half of antennal segments III–VII with lines of sculpture in *comosus*) ; (2) postocular setae with expanded apex (whereas *comosus* with pointed postocular setea); (3) abdominal tergites II to VII each with two pairs of wing-retaining setae (only one pair of wing-retaining setae on these segments in *comosus*) ; (4) male’s pore plate on abdominal sternite VIII narrow and slightly arched, occasionally disconnected (whereas pore plate with wider band which reaches lateral margins in *comosus*).

#### Psalidothrips armatus

Taxon classificationAnimaliaThysanopteraPhlaeothripidae

Okajima

Fig. 12

Psalidothrips armatus Okajima, 1983: 6.

##### Comments.

This species (Fig. 12) belongs to the group in which the fore tarsus is armed with a tooth in both sexes. Wang et al. (2007) recorded a male of this species in Hainan, China. In this study, we examined the male specimen labelled as *P. armatus* by Zhang and Tong (1997) and considered that this single male specimen seems to be an unknown species which is similar to the male of *P. angustus* sp. n. or *P. longidens.* In China, this species so far is only recorded in Hainan.

##### Distribution.

China (Hainan); Thailand.

#### Psalidothrips ascitus

Taxon classificationAnimaliaThysanopteraPhlaeothripidae

(Ananthakrishnan)

Figs 13–14 , 71

Callothrips ascitus Ananthakrishnan, 1969: 176.
Psalidothrips ascitus (Ananthakrishnan): Okajima, 1983: 6.

##### Comments.

This species is found widely across the tropical and subtropical areas of China, also in other parts of the world. It is most closely related to *P. lewisi* (Bagnall). Their males are difficult to distinguish from each other as their pore plates are very similar in shape (Figs 14, 71, 76), but the females can be distinguished from those of *P. lewisi* by the length of the postocellar setae that are usually as long as the diameter of an ocellus or shorter, and the colour of the antennae and head that are almost uniformly brown (Fig. 13) (Okajima 2006). However, these two species were always collected together at the same sampling sites from leaf litter. Overall, in structure these two species are very similar to each other and the possibility exists that they may represent a single, widespread species.

##### Distribution.

China (Hubei, Guizhou, Hunan, Jiangxi, Yunnan, Guangdong, Hainan, Taiwan); Japan; Malaysia; India.

#### Psalidothrips bicoloratus

Taxon classificationAnimaliaThysanopteraPhlaeothripidae

Wang, Tong & Zhang

Figs 15–16 , 72

Psalidothrips bicoloratus Wang, Tong & Zhang, 2007: 28.

##### Comments.

This species is very similar to *P. amens* in general appearance. However, it is distinguished from the latter by the following characters: head and antennal segments I–II, V–VIII brown, rest of body yellow (Fig. 15–16); postocular setae shorter than eyes, cheeks straight and weakly constricted behind eyes.

##### Distribution.

China (Guangdong).

#### Psalidothrips chebalingicus

Taxon classificationAnimaliaThysanopteraPhlaeothripidae

Zhang & Tong

Figs 17–18 , 62–66 , 73

Psalidothrips chebalingicus Zhang & Tong, 1997: 87.

##### Diagnosis.

This species was originally described in Chinese with the male as holotype (Zhang & Tong 1997). Unfortunately, in original paper the illustrations were distorted by compression in the process of printing and the female was described very briefly, which meant that the female of *P. chebalingicus* could be confused with other similar species. The diagnosis is emended as follows:

**Female macropterous** (Fig. 17). Body yellow except head, antennal segments I–II and VI–VIII (Fig. 66), margins of pterothorax brown; abdominal tergite II brown with median portion yellow; antennal segments III–V yellowish brown but gradually darkened distally. Head (Fig. 62) slightly wider than long, dorsal surface smooth; cheeks weakly swollen and constricted just behind eyes; postocellar setae slightly longer than hind ocellus; postocular setae slightly longer than eyes and pointed at apex. Antennae 8-segmented (Fig. 66), surface without sculpture; segments III–IV each with two sense cones; segment VIII slightly longer than segment VII. Maxillary stylets reaching approximately half distance to postocular setae and far apart, V-shaped. Pronotum dorsal surface smooth, am and aa minute, ml apex blunt, epim and pa pointed at apex (Fig. 63). Fore tarsal tooth absent. Pelta hat-shaped, weakly sculptured on anterior half, with a pair of campaniform sensilla posteriorly (Fig. 64); abdominal tergites II–VII with two pairs of weakly sigmoid wing-retaining setae; abdominal tergite IX setae S1 subequal to tube in length (Fig. 65), setae S2 longer than tube, both S1 and S2 pointed at apex.

**Male macropterous** (Fig. 18). Similar in colour and structure to females, but body smaller; fore tarsal tooth present; pore plate on abdominal sternite VIII narrow and slightly arched medially (Fig. 73); abdominal tergite IX setae S1 slightly shorter than tube but much longer than S2.

##### Comments.

This species is somewhat similar to *P. ascitus* in colour and structure. However, it can be distinguished from the latter by the following main features: antennal segments III–IV each with two sense cones; pore plate on abdominal sternite VIII located medially, not reaching lateral margin.

##### Distribution.

China (Hunan, Guangdong).

#### Psalidothrips comosus sp. n.

Taxon classificationAnimaliaThysanopteraPhlaeothripidae

http://zoobank.org/5ADD2A6A-82F9-4603-BEE8-4E794C91B985

Figs 3–4 , 37–44 , 68

##### Material examined.

**Holotype** female (macropterous): **CHINA. Guangdong**: Shenzhen City, Honghu Park (22°33'N, 114°07'E), collected from leaf litter of *Araucaria heterophylla* (Araucariaceae), 2.xi.2017 (Chao Zhao).

##### Paratypes.

13 macropterous females, 4 apterous females, and 6 apterous males, collected with holotype. Nineteen apterous females, 5 macropterous females, and 8 apterous males, collected at the same locality as holotype, 30.x.2014 (Chao Zhao).

##### Description.

**Female macropterous** (Fig. 3). Body yellow except for head, anterior and lateral margins of pterothorax, abdominal segment II dark brown; abdominal tergites III–VIII yellow with light brown shadings laterally; tube yellow with light brown basally. Wings shaded with greyish brown.

*Head* (Fig. 37) wider than long, faintly sculptured on posterior margin; cheeks almost straight or slightly widened towards base, but constricted behind eyes; eyes approximately 1/3 of head length; postocellar setae long and acute, approximately 1.5–2.0 times longer than the diameter of hind ocellus; postocular setae long and acute, approximately 2.0 times longer than eyes. Antennae 8-segmented (Fig. 44) and slightly moniliform, apical half of antennal segments III–VII with lines of sculpture; segment III vasiform, IV–V globular, segment VIII distinctly longer than segment VII; segments III–IV with 3 and 4 sense cones respectively. Maxillary stylets reaching approx. half way to postocular setae and wide apart.

*Pronotum* (Fig. 38) broad, surface smooth with a weak median longitudinal line; three pairs of major setae (ml, epim, pa) well developed, elongate and acute, aa setae fine and long, slightly shorter or subequal to interocular setae in length. Mesopresternum eroded with small irregular sclerites laterally. Fore wing sub-basal setae S1 shortest, S2 longer than S3. Fore tarsal tooth present (Fig. 39).

*Pelta* irregularly hat-shaped (Fig. 41), sculptured on anterior half, campaniform sensilla absent in holotype. Abdominal tergites II to VII each with one pair of straight wing-retaining setae (Fig. 42); tergite IX setae S1 and S2 long and acute, setae S1 as long as or slightly longer than S2, setae S2 longer than tube; tube slightly longer than head.

**Female apterous**. Similar to macropterous female in structure, but eyes smaller, approximately 1/4 of head length; postocellar setae approximately 2.5–3.5 times longer than diameter of hind ocellus; postocular setae elongate and acute, approximately 2.5 times longer than eyes.

*Measurements* (holotype female in microns). Distended body length 1680. Head length 155, width 178; eye length 60; postocular setae length 120; diameter of posterior ocellus 20; postocellar setae length 50. Antennal length 360, segments I–VIII length (width) as follows: 35 (39); 45 (30); 57 (33); 40 (35); 37 (39); 40 (35); 35 (27); 55 (20). Pronotum median length 135, median width 260; length of major setae: aa 50, ml 90, pa 130, epim 95. Abdominal tergite IX S1 setae length 212, S2 setae length 200. Tube length 165, basal width 80, apical width 40; anals 100.

**Male apterous** (Fig. 4). Colour and chaetotaxy similar to apterous females, but femora thickened and fore tarsal tooth well developed (Fig. 40); pore plate on abdominal sternite VIII narrow and arched, nearly reaching lateral margins (Fig. 68); tergite IX setae S1 much longer than S2; setae S2 shorter than tube (Fig. 43).

*Measurements* (paratype male in microns). Distended body length 1620. Head length 165, width 170; eye length 40; postocular setae length 100; diameter of posterior ocellus 10; postocellar setae length 25. Antennal length 340, segments I–VIII length (width) as follows: 37 (38); 40 (28); 55 (28); 38 (32); 36 (33); 38 (30); 35 (25); 47 (17). Pronotum median length 125, median width 255; length of major setae: aa 35, ml 87, pa 120, epim 80. Abdominal tergite IX S1 setae length 135, S2 setae length 85. Tube length 125, basal width 70, apical width 33; anals 100.

##### Etymology.

Specific epithet from Latin *comosus* which means long haired, and refers to the new species having relatively long body setae.

##### Distribution.

China (Guangdong).

##### Comments.

This new species is similar to *P. taylori* Mound & Walker from Australia and New Zealand in sharing the elongate postocular setae, distinct pronotal aa setae, and only one pair of wing-retaining setae on abdominal tergites (Mound and Walker 1986). However, it can be readily distinguished from the latter by the antennal segments III–VII with lines of sculpture, segments III–IV with three and four sense cones respectively, and a fore tarsal tooth present in both sexes. Campaniform sensilla are absent from the pelta of the holotype, but are present on the pelta of paratypes from the same population. This new species is also similar to the new species, *P. angustus*, as discussed above.

#### Psalidothrips consimilis

Taxon classificationAnimaliaThysanopteraPhlaeothripidae

Okajima

Figs 19–20 , 74

Psalidothrips consimilis Okajima, 1992: 541.

##### Material examined.

**CHINA. Guangdong**: Foshan City, Suoluo Nature Reserve (22°29'N,111°30'E), 2 females and 1 male, 27.iii.2005 (Jun Wang), 2 females and 1 male, 3.vii.2014 (Chao Zhao).

##### Distribution.

China (Guangdong); Japan (Ryukyu Islands).

##### Comments.

Described originally from Ryukyu Islands, Japan (Okajima 1992), this thrips is here newly recorded from China. In the description (Okajima 1992), the postocellar setae are minute, usually shorter than the diameter of the hind ocellus, but in the specimens listed here these setae are variable in length: some of them are much longer than the hind ocellus. The female (Fig. 19) is very similar to that of *P. ascitus* (Okajima 1992, 2006). However, the males (Fig. 20) can be easily distinguished from *P. ascitus* by the narrow and incomplete pore plate on abdominal sternite VIII (Fig. 74).

#### Psalidothrips elagatus

Taxon classificationAnimaliaThysanopteraPhlaeothripidae

Wang, Tong & Zhang

Figs 21–22 , 75

Psalidothrips elagatus Wang, Tong & Zhang, 2007: 26.

##### Comments.

Wang and Tong (2007) stated that this species has two sense cones on antennal segment III, but re-examination of the type material has found this segment to have three sense cones, although the ventral one is small and short. Therefore, *P. elagatus* belongs to the group that have antennal segments III and IV each with three sense cones. This species (Figs 21–22) is similar to *P. bicoloratus*, but can be distinguished by the key above.

##### Distribution.

China (Guangdong).

#### Psalidothrips fabarius sp. n.

Taxon classificationAnimaliaThysanopteraPhlaeothripidae

http://zoobank.org/6CB4F657-6301-4695-9E8D-95D17CB66609

Figs 5–6 , 45–49 , 69

##### Materials examined.

**Holotype** female: **CHINA. Guangdong**: Guangzhou City, Arboretum of South China Agricultural University (23°09'N, 113°21'E), 29.xii.2013 (Jingna Li).

##### Paratypes.

Ten females and 3 males, collected with holotype; same locality as holotype: 2 females, 31.iii.2011 (Tao Song), 1 female and 1 male, 26.viii.2013 (Jingna Li), 1 female and 1 male, 29.xii.2013 (Jingna Li), 3 females and 1 male, 26.vii.2014 (Chao Zhao), 6 females and 3 males, 14.vii.2014 (Chao Zhao); Guangzhou City, Huolushan Forest Park (23°10'47"N, 113°22'44"E), 2 females and 2 males, 1.vi.2014 (Jingna Li).

##### Description.

**Figures 5–8.**
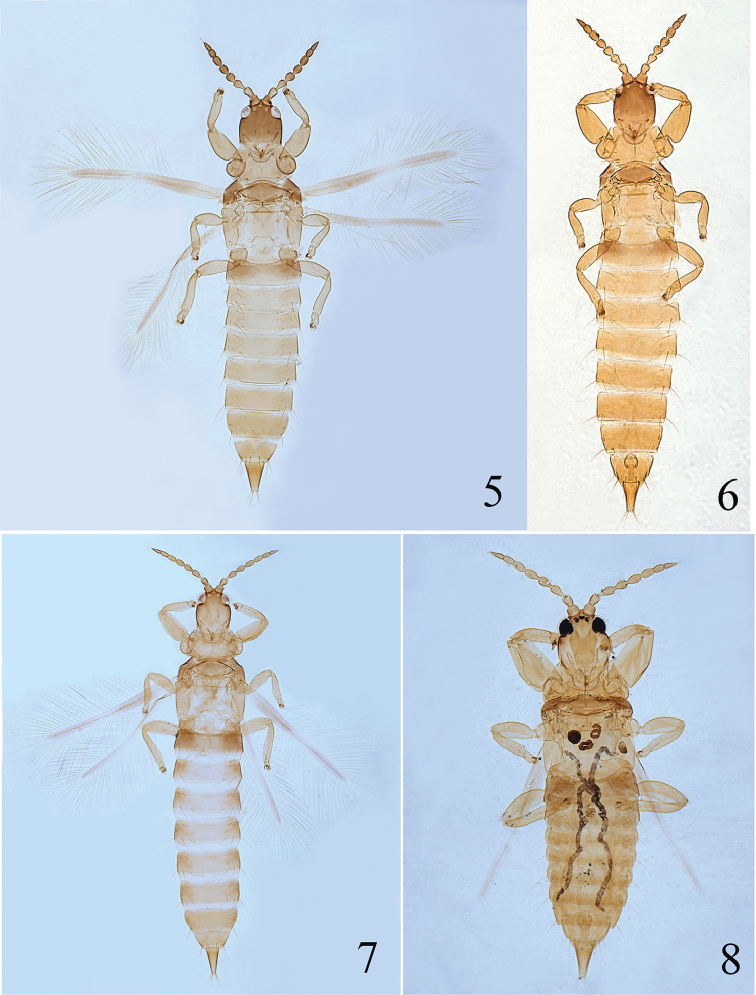
New *Psalidothrips* species. *Psalidothrips fabarius* sp. n.: **5** female **6** male; *Psalidothrips latizonus* sp. n. **7** female **8** male.

**Female macropterous** (Fig. 5). Body yellow except head, mesothorax, abdominal tergite II brown, the rest of body yellow. Antennal segments I and basal half of II light brown, segment III yellow, IV–VIII yellowish brown gradually darkened distally. Wings shaded with greyish brown but paler medially.

*Head* (Fig. 45) almost as long as wide, dorsal surface smooth, faintly sculptured at base; cheeks weakly swollen, slightly constricted just behind eyes. Eyes one-third as long as head; postocular setae approximately 1.5 times longer than eyes and weakly expanded at apex; postocellar setae approximately twice as long as hind ocellus or longer. Antennae 8-segmented, somewhat moniliform, surface without sculpture (Fig. 49); segment VIII longer than segment VII; segments III and IV each with two sense cones. Maxillary stylets reaching about one-third way to postocular setae and wide apart, often V-shaped.

*Pronotum* (Fig. 46) broad, surface smooth with a weak median longitudinal line; pronotal am and aa minute, the other three pairs of major setae well developed, pa longest, ml slightly longer than epim, epim and pa pointed, but ml expanded at apex. Fore tarsal tooth absent. Sub-basal wing seta S1 minute, S2 longer than S3, both pointed at apex.

*Pelta* nearly bell-shaped with short lateral lobes (Fig. 47), anterior half distinctly sculptured, a pair of campaniform sensilla present. Abdominal tergites II–VII with two pairs of simply curved wing-retaining setae; S1 and S2 on tergite IX subequal in length (Fig. 48), slightly shorter than tube, all pointed at apex; basal width of tube approximately 3.0 times wider than apical width; anal setae much shorter than tube.

*Measurements* (holotype female in microns). Body length 1680. Head length 180, head maximum width 175; eye length 55, postocular setae length 80; diameter of posterior ocellus 16, postocellar setae length 32. Antennal length 360, segments I–VIII length (width) as follows: 42(42); 42 (33); 56 (35); 46 (35); 50 (32); 46 (31); 33 (27); 42 (19). Pronotum median length 145, median width 240; length of major setae: ml 65, pa 80, epim 60. Fore wing length 720, subbasal setae S1–S3 length: 4, 35, 23. Abdominal tergite IX S1 setae length 100, S2 setae length 95. Tube length 120, tube basal width 70, apical width 23; anals 100.

**Male apterous** (Fig. 6). Similar to female in structure and colour, but smaller and fore tarsus armed with a tooth; setae S2 much shorter than S1 on abdominal tergite IX; abdominal sternite VIII pore plate arch-shaped with a projection medially (Fig. 69).

*Measurements* (paratype male in microns). Distended body length 1370. Head length 150, width 140; eye length 40; postocular setae length 68; diameter of posterior ocellus 12; postocellar setae length 28. Antennal length 295, segments I–VIII length (width) as follows: 32 (42); 35 (30); 46 (30); 35 (32); 35 (30); 38 (28); 32 (26); 42 (19). Pronotum median length 130, median width 210; length of major setae: ml 44, pa 64, epim 44. Abdominal tergite IX S1 setae length 85, S2 setae length 60. Tube length 100, basal width 55, apical width 25; anals 60.

##### Distribution.

China (Guangdong).

##### Etymology.

The specific epithet, *fabarius*, is from the Latin word meaning bead-like, referring to the moniliform antennal segments.

##### Comments.

This new species appears to be most similar in appearance to *P. ochraceus* Okajima from Ryukyu Islands, Japan, particularly in having two sense cones on antennal segments III and IV, and the elongate postocellar setae, but it can be readily distinguished from the latter by the following characteristics: (1) postocular setae expanded at apex (pointed in *ochraceus*); (2) pronotal ml setae expanded at apex (ml pointed in *ochraceus*); (3) pelta distinctly sculptured on anterior half (whereas pelta indistinctly sculptured in *ochraceus*); (4) abdominal tergite IX setae S1 and S2 subequal in length, but shorter than tube (whereas in *ochraceus*, S1 slightly shorter than tube, S2 longer than tube); (5) three sub-basal wing setae present on fore wing (only one minute sub-basal wing seta present in *ochraceus*).

#### Psalidothrips latizonus sp. n.

Taxon classificationAnimaliaThysanopteraPhlaeothripidae

http://zoobank.org/25FD6569-3677-4870-A161-00EFA248D28E

Figs 7–8 , 50–54 , 70

##### Material examined.

**Holotype: CHINA. Hainan**: 1 female, Ledong County, Jianfengling National Nature Reserve (18°44'N, 108°51'E), 30.x.1986 (Xiaoli Tong).

##### Paratypes.

Two females 2 males, same data as holotype. **Guangdong**: 1 male, Haifeng County, Mt. Lianhuashan (23°03'N, 115°15'E), 14.ix.2005 (Jun Wang).

**Female macropterous** (Fig. 7). Head largely yellow or yellowish brown with dark brown margins; mesonotum yellowish brown with dark brown margin, abdominal tergite II brown, darker than other tergites, abdominal segments yellow shaded with pale brown laterally, the rest of body yellow. Antennal segments I–II pale brown, III–VIII shading gradually from yellow to pale brown towards apex. Wings shaded with greyish brown but paler medially.

*Head* (Fig. 50) almost as long as broad, dorsal surface smooth with a few lines of sculpture posteriorly; cheeks slightly swollen and constricted just behind eyes. Eyes approximately one-third of head length; postocellar setae approximately 2.5 times longer than hind ocellus; postocular setae bluntly acute, as long as or slightly longer than eyes . Antennae 8-segmented (Fig. 54), surface without sculpture; segments III and IV each with two sense cones, segment VIII longer than segment VII. Maxillary stylets short and wide apart, often V-shaped.

*Pronotum* about 0.8 times as long as head, almost smooth (Fig. 51); ml and epim subequal in length, pa longest, all bluntly acute. Fore tarsal tooth absent. Fore wing sub-basal wing seta S1 minute, S2 longer than S3, both pointed at apex.

*Pelta* nearly hat-shaped (Fig. 52), faintly sculptured, with a pair of campaniform sensilla. Abdominal tergites II to VII each with two pairs of sigmoid wing-retaining setae; tergite IX setae S1 shorter than S2 which slightly longer than tube (Fig. 53); basal width of tube 3–4 times wider than apical width.

*Measurements* (holotype female in microns). Distended body length 2050. Head length 205, width 203; eye length 70; postocular setae length 90; diameter of posterior ocellus 16; postocellar setae length 32. Antennal total length 425, segments I–VIII length (width): 42 (39); 47 (28); 63 (30); 57 (30); 59 (28); 59 (25); 43 (23); 55 (17). Pronotum median length 160, median width 260; length of major setae: ml length 45, pa length 65, epim length 50. Fore wing length 800, subbasal setae S1–S3 length: 3, 15, 13. Abdominal tergite IX setae S1 length 130, S2 length 165. Tube length 145, tube basal width 80, apical width 27, anals 100.

**Male macropterous** (Fig. 8). Similar in colour and structure to female except for fore tarsal tooth present and setae S1 slightly longer than S2 on abdominal tergite IX; pore plate on abdominal sternite VIII broadly arched (Fig. 70).

*Measurements* (paratype male in microns). Distended body length 1230. Head length 190, width 175; eye length 55, postocular setae length 75; diameter of posterior ocellus 15; postocellar setae length 38. Antennal total length 370, segments I–VIII length (width): 35 (40); 36 (28); 52 (25); 45 (29); 44 (25); 48 (28); 34 (24); 45 (20). Pronotum median length 140, median width 240; length of major setae: ml length 45, pa length 65, epim length 50. Abdominal tergite IX setae S1 length 105, S2 length 85. Tube length 115, basal width 67, apical width 22, anals 85.

##### Distribution.

China (Guangdong, Hainan).

##### Etymology.

The specific epithet, *latizonus*, is from the Latin adjective meaning broad band, in reference to the broad male pore plate.

##### Comments.

The new species is closely similar to *P. chebalingicus* in general appearance, but differs from it as follows: head largely yellowish brown but darkened laterally (head uniformly brown in *chebalingicus*); antennal segments I–II pale brown, III–VIII shading gradually from yellowish brown to pale brown towards apex (antennae yellow except segments I–II and VI–VIII brown in *chebalingicus*); postocellar setae about 2.5 times as long as hind ocellus (postocellar setae slightly longer than hind ocellus in *chebalingicus*); antennal segment VIII longer than segment VII (antennal segment VIII as long as segment VII in *chebalingicus*). Moreover, the males have the broad pore plate reaching the lateral margin of sternite VIII, whereas the pore plate of *P. chebalingicus* is narrow and slightly arched, not reaching the lateral margin.

#### Psalidothrips lewisi

Taxon classificationAnimaliaThysanopteraPhlaeothripidae

(Bagnall)

Figs 23–24 , 76

Trichothrips lewisi Bagnall, 1914: 30.
Psalidothrips alaris Haga, 1973: 76. Synonymised by Okajima and Urushihara 1992: 167.
Psalidothrips lewisi (Bagnall): Okajima and Urushihara 1992: 167.

##### Comments.

This species has a wide geographical range from Shandong province to Hainan province in China. The Chinese specimens listed here have been compared with the Japanese specimens (provided by S. Okajima) and, despite antennal segments III–VIII of the Japanese specimens being almost uniformly yellow and much paler than the Chinese specimens (Figs 23–24), they are considered to represent *P. lewisi*. Moreover, the macropterous form in both sexes is more common than the apterous form among the Chinese specimens.

##### Distribution.

China (Shandong, Guizhou, Hunan, Jiangxi, Yunnan, Guangdong, Hainan); Japan.

#### Psalidothrips longidens

Taxon classificationAnimaliaThysanopteraPhlaeothripidae

Wang, Tong & Zhang

Figs 25–26 , 77

Psalidothrips longidens Wang, Tong & Zhang, 2007: 30.

##### Comments.

This species belongs to the group in which the fore tarsal tooth is present in both sexes (Figs 25–26). The following characters can distinguish it from congeneric species: antennal segments III and IV each with three sense cones; fore tarsus of females armed with a long and strong tooth, pelta irregularly rectangular without campaniform sensilla, and male with a transverse and weakly curved pore plate (Fig. 77) which does not reach the lateral margins.

##### Distribution.

China (Guangdong).

#### Psalidothrips nigroterminatus sp. n.

Taxon classificationAnimaliaThysanopteraPhlaeothripidae

http://zoobank.org/2703C57E-AD67-4E79-A282-EC10A3E10123

Figs 9–10 , 55–61

##### Material examined.

**Holotype: CHINA. Hainan**: 1 female, Qiongzhong County, Limushan National Forest Park (19°12'40"N, 113°12'39"E, alt. 1200m), 24.x.2017 (Chao Zhao).

##### Paratypes.

Four females and 1 male, collected with holotype. **Yunnan**: One female and 1 male, Mengla County (21°56'N, 101°15'E), from leaf litter of bamboo, 3.x.2010 (Nie Jing & Sun Jun); 13 females and 10 males (preserved in the Insect Collection, Yunnan Agricultural University, YAU), Mengla County (21°56'N, 101°15'E), collected from leaf litter of bamboo, 3.x.2010 (Nie Jing & Sun Jun).

**Figures 9–10.**
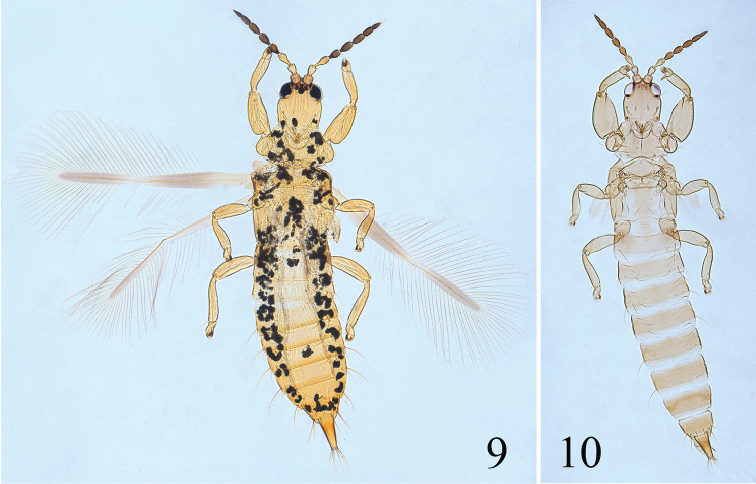
*Psalidothrips nigroterminatus* sp. n.: **9** female **10** male.

**Female macropterous** (Fig. 9). *Body* largely yellow, head yellow tinged with light brown anteriorly, abdominal segments shading gradually from yellow to yellowish brown towards tube; antennal segments I–III yellow, IV–VIII dark brown. Wings shaded greyish brown but paler medially.

**Figures 11–28.**
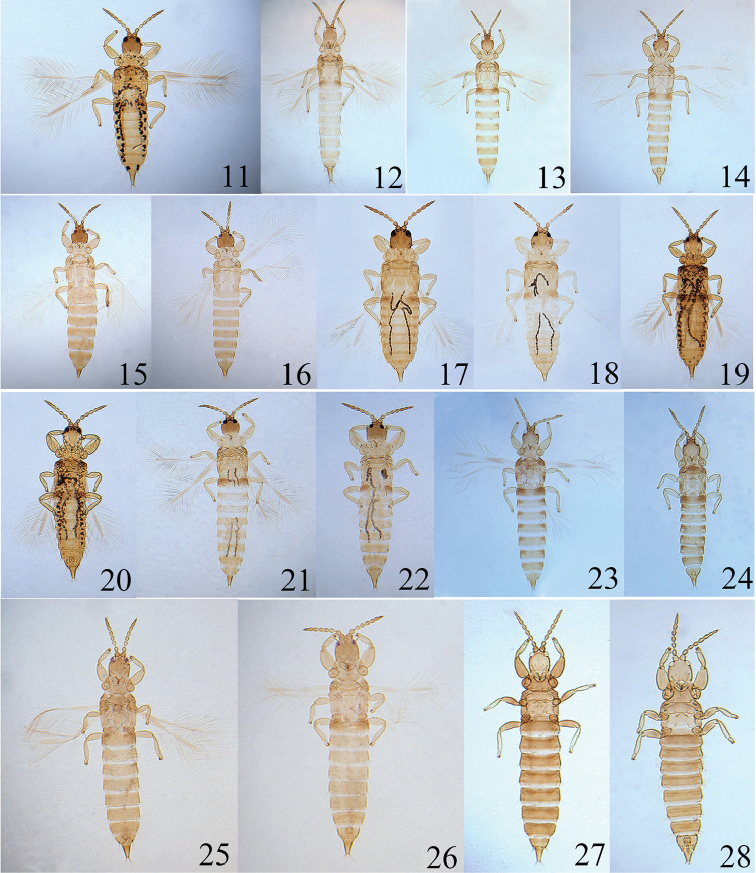
*Psalidothrips* species. *P. amens* **11** female; *P. armatus* **12** female; *P. ascitus* **13** female **14** male; *P. bicoloratus* **15** female **16** male; *P. chebalingicus* **17** female **18** male; *P. consimilis* **19** female **20** male; *P. elagatus* **21** female **22** male; *P. lewisi* **23** female **24** male; *P. longidens* **25** female **26** male; *P simplus* **27** female **28** male.

**Figures 29–36.**
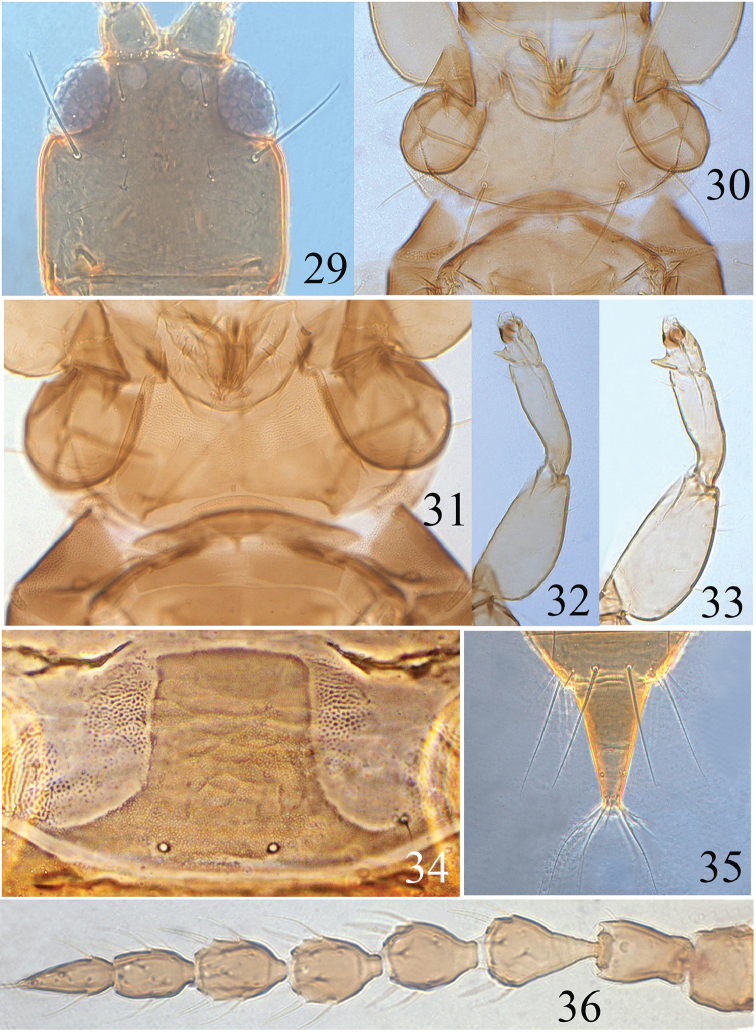
*Psalidothrips angustus* sp. n. **29** head **30** pronotum **31** ventral view of prothorax **32** fore leg of female **33** fore leg of male **34** pelta **35** tube **36** antenna.

**Figures 37–44.**
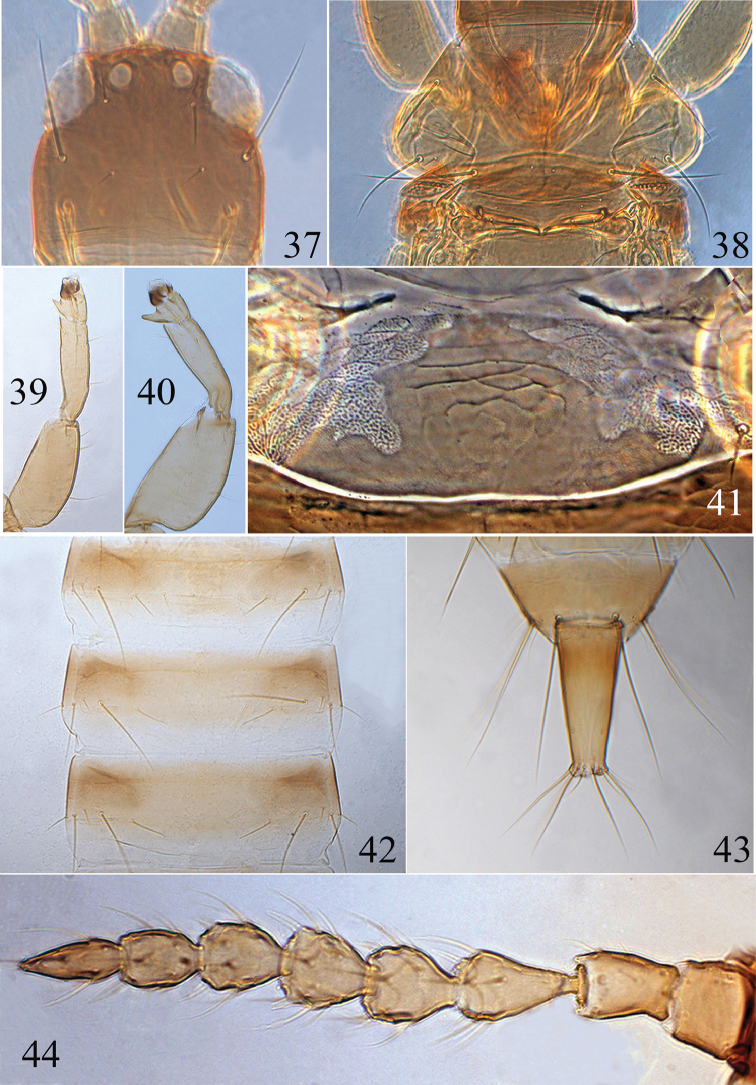
*Psalidothrips comosus* sp. n. **37** head **38** pronotum **39** fore leg of female **40** fore leg of male **41** pelta **42** abdominal tergites III–V **43** tube **44** antenna.

**Figures 45–49.**
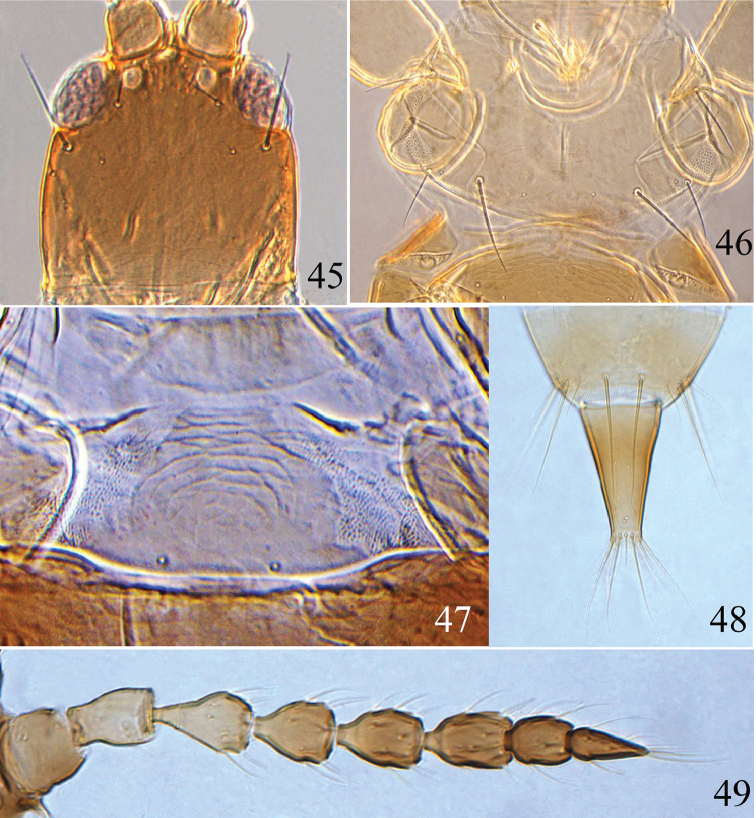
*Psalidothrips fabarius* sp. n. **45** head **46** pronotum **47** pelta **48** tube **49** antenna.

**Figures 50–54.**
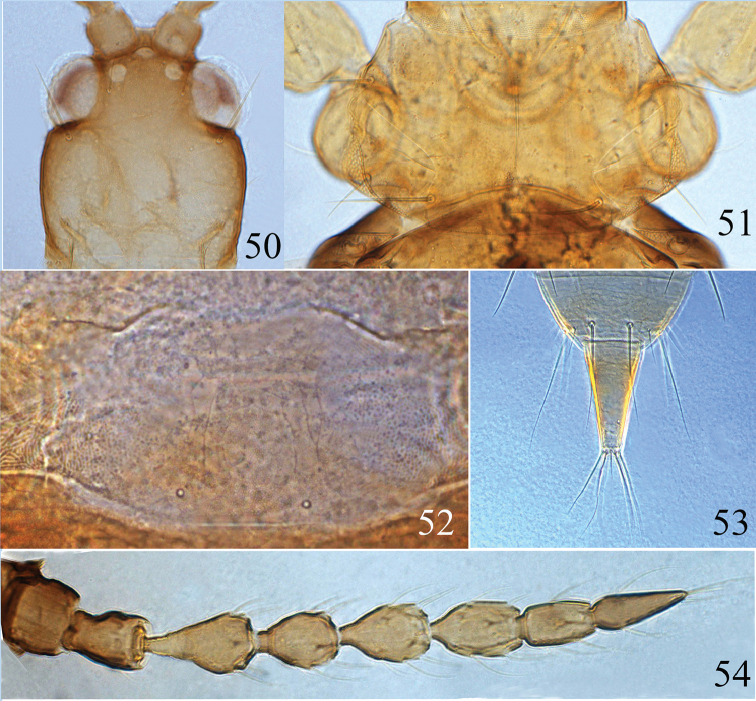
*Psalidothrips latizonus* sp. n. **50** head **51** pronotum **52** pelta **53** tube **54** antenna.

**Figures 55–61.**
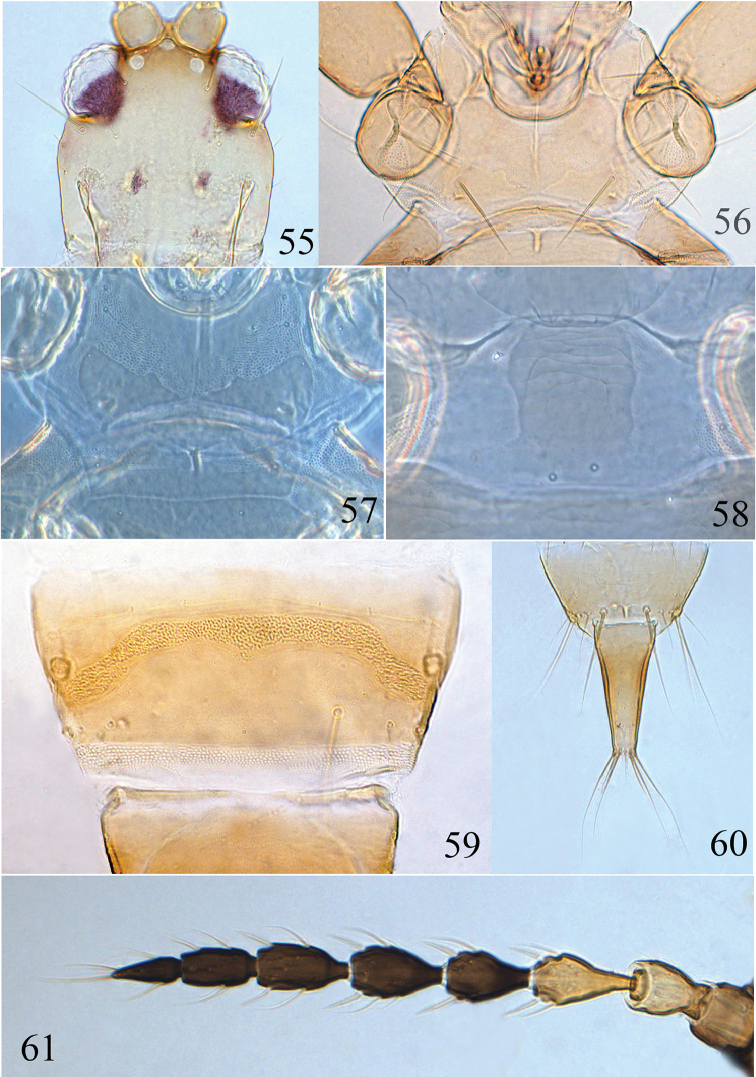
*Psalidothrips nigroterminatus* sp. n. **55** head **56** pronotum **57** ventral view of prothorax **58** pelta **59** male pore plate **60** tube **61** antenna.

**Figures 62–66.**
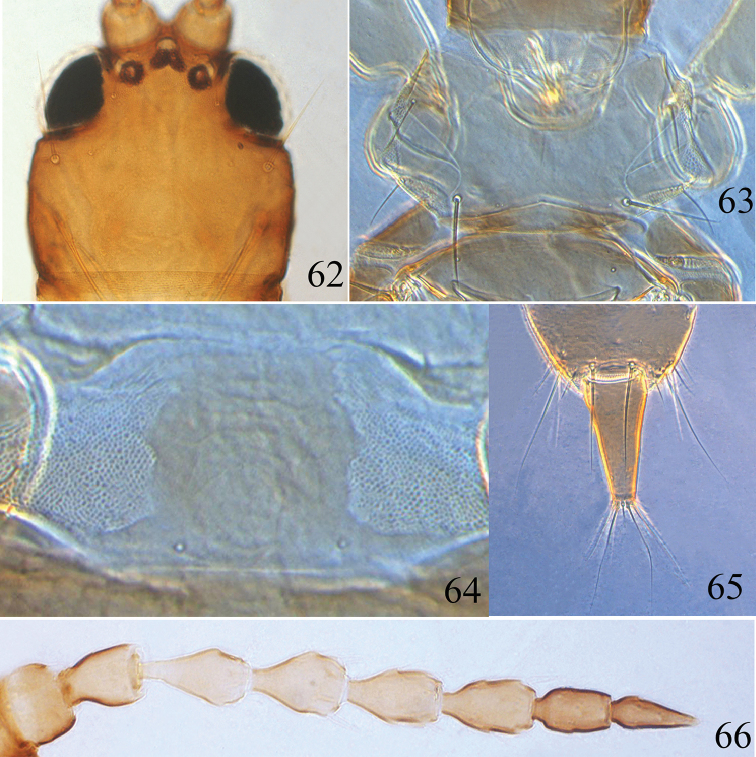
*Psalidothrips chebalingicus* Zhang & Tong **62** head **63** pronotum **64** pelta **65** tube **66** antenna.

**Figures 67–70.**
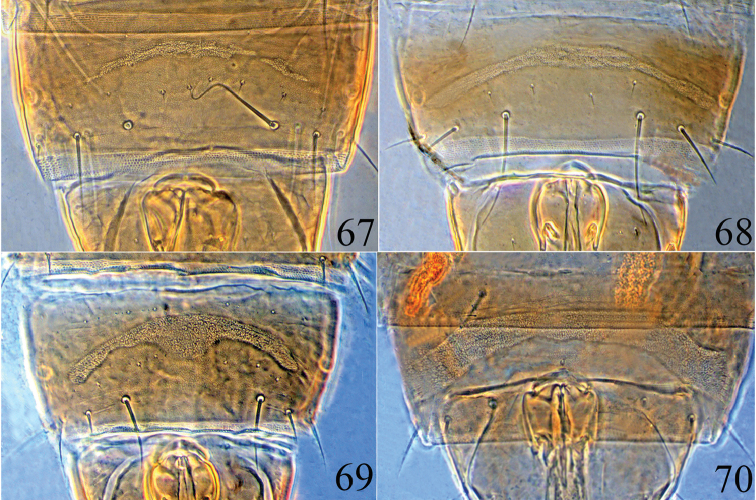
Male pore plate of *Psalidothrips* species: **67** *P. angustus* sp. n. **68** *P. comosus* sp. n. **69** *P. fabarius* sp. n. **70** *P. latizonus* sp. n.

**Figures 71–78.**
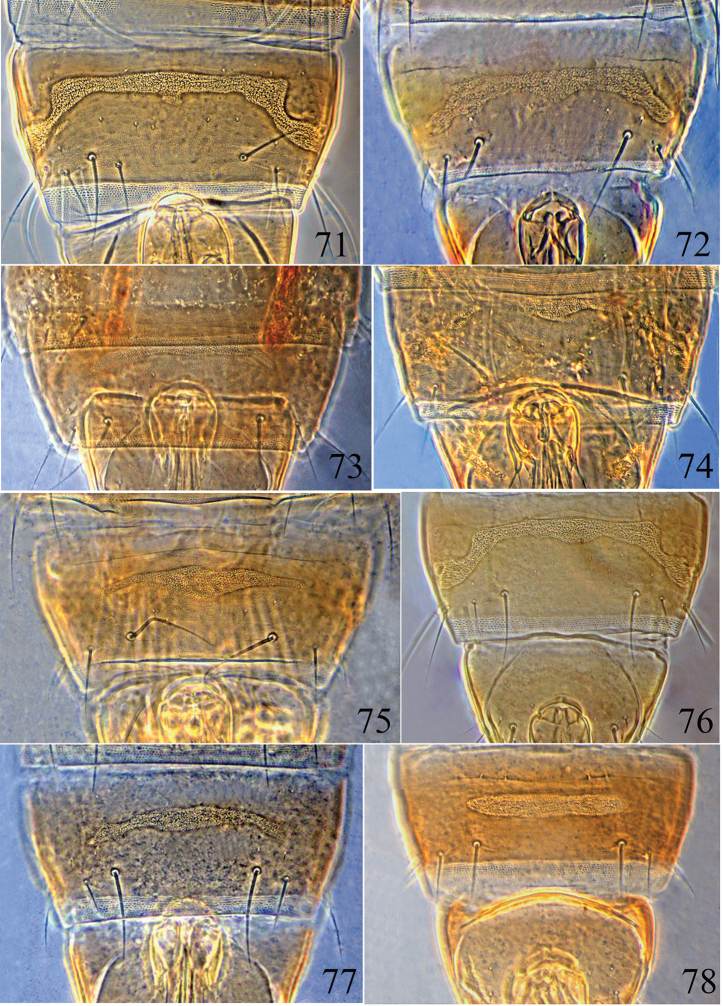
Male pore plate of *Psalidothrips* species: **71** *P. ascitus* **72** *P. bicoloratus* **73** *P. chebalingicus* **74** *P. consimilis* **75** *P. elagatus* **76** *P. lewisi* **77** *P. longidens* **78** *P. simplus*

*Head* (Fig. 55) wider than long, faintly sculptured on posterior margin; cheeks slightly swollen and constricted behind eyes; eyes approximately 2/5 of head length; postocellar setae long and acute, approximately 2.5 times longer than diameter of hind ocellus; postocular setae blunt or weakly expanded at apex, as long as or slightly longer than eyes. Antennae 8-segmented (Fig. 61), surface without sculpture; antennal segments III and IV each with two sense cones, segment VIII shorter than segment VII. Maxillary stylets reaching about half way to postocular setae and wide apart.

*Pronotum* broad (Fig. 56), surface smooth with a median longitudinal line; three pairs of major setae well developed, pa longest and acute at apex; ml subequal to epim in length, both slightly expanded apically. Fore tarsal tooth absent. Sub-basal wing seta S1 minute, S2 longer than S3, both pointed at apex. Mesonotum weakly sculptured on anterior third; metanotum smooth with longitudinal sculpture laterally. Mesopresternum complete and boat-shaped (Fig. 57).

*Pelta* (Fig. 58) hat-shaped with a pair of campaniform sensilla posteriorly, surface sculptured on anterior half. Abdominal tergites II to VII each with two pairs of wing-retaining setae; tergite IX setae S1 and S2 pointed (Fig. 60), S1 shorter than S2 which are subequal to tube in length; basal width of tube approximately 2.5 times wider than apical width.

*Measurements* (holotype female in microns). Distended body length 1810. Head length 170, width 170; eye length 67; postocular setae length 67; diameter of posterior ocellus 12; postocellar setae length 30. Antennal total length 365, segments I–VIII length (width): 36 (40); 44 (32); 56 (28); 44 (30); 49 (33); 51 (25); 44 (21); 41 (16). Pronotum median length 130, median width 250; length of major setae: ml length 42, pa length 75, epim length 42. Fore wing length 710, subbasal setae S1–S3 length: 3, 15, 3. Abdominal tergite IX setae S1 length 110, S2 length 130. Tube length 130, tube basal width 62, apical width 25, anals 125.

**Male micropterous** (Fig. 10). Similar in colour and structure to female except for fore tarsal tooth present; pore plate on abdominal sternite VIII arched, slightly straight anteriorly and reaching lateral margins (Fig. 59).

*Measurements* (paratype male in microns). Distended body length 1640. Head length 160, width 160; eye length 60, postocular setae length 60; diameter of posterior ocellus 12; postocellar setae length 32. Antennal total length 335, segments I–VIII length (width): 33 (35); 39 (26); 52 (24); 43 (24); 44 (24); 45 (23); 41 (19); 39 (14). Pronotum median length 140, median width 230; length of major setae: ml length 48, pa length 70, epim length 43. Abdominal tergite IX setae S1 length 100, S2 length 85. Tube length 105, basal width 56, apical width 22, anals 100.

##### Distribution.

China (Yunnan, Hainan).

##### Etymology.

The species name is an arbitrary combination of two Latin adjectives, *niger* meaning black and *terminatus* meaning terminal, in reference to the antennae with dark brown distal segments.

##### Comments.

The new species belongs to the group in which antennal segments III and IV both have two sense cones. It can be distinguished from the other members of the group by the following combination of features: (1) body largely yellow but antennal segments IV–VIII dark brown; (2) mesopresternum complete and boat-shaped; (3) pronotal posteroangular setae acute at apex and much longer than other major pronotal setae; (4) abdominal tergite II concolourous with the other tergites, and (5) male pore plate arched but slightly straight anteriorly.

#### Psalidothrips simplus

Taxon classificationAnimaliaThysanopteraPhlaeothripidae

Haga

Figs 27–28 , 78

Psalidothrips simplus Haga, 1973: 77.

##### Comments.

This species (Figs 27–28) is easily separated from congeneric species by the following combination of characters: body largely yellowish brown, abdominal tergite II almost concolourous with other tergites; abdominal tergites III to VII each with one pair of simple wing-retaining setae; pelta broad hat-shaped or trapezoidal and male with a transversely long oval pore plate (Fig. 78). Okajima (2006) pointed out that antennal segment III always has two sense cones, but those on segment IV are variable in number from two to four, which is the same in the Chinese specimens.

##### Distribution.

China (Hubei, Guizhou, Hunan, Jiangxi, Yunnan, Guangdong, Hainan); Japan.

## Supplementary Material

XML Treatment for Psalidothrips amens

XML Treatment for Psalidothrips angustus

XML Treatment for Psalidothrips armatus

XML Treatment for Psalidothrips ascitus

XML Treatment for Psalidothrips bicoloratus

XML Treatment for Psalidothrips chebalingicus

XML Treatment for Psalidothrips comosus

XML Treatment for Psalidothrips consimilis

XML Treatment for Psalidothrips elagatus

XML Treatment for Psalidothrips fabarius

XML Treatment for Psalidothrips latizonus

XML Treatment for Psalidothrips lewisi

XML Treatment for Psalidothrips longidens

XML Treatment for Psalidothrips nigroterminatus

XML Treatment for Psalidothrips simplus
